# Self-Reported Ache, Pain, or Numbness in Feet and Use of Computers amongst Working-Age Finns

**DOI:** 10.3390/healthcare4040082

**Published:** 2016-11-07

**Authors:** Leena Korpinen, Rauno Pääkkönen, Fabriziomaria Gobba

**Affiliations:** 1 Clinical Physiology and Neurophysiology Unit, North Karelia Central Hospital and Honkalampi Centre, Joensuu FIN-80210, Finland; 2 TMI Rauno Pääkkönen, Timpurinkatu 7, Tampere FIN-33720, Finland; rauno.paakkonen@gmail.com; 3 Department of Biomedical, Metabolic and Neural Sciences, University of Modena and Reggio Emilia, Via Campi 287, Modena 41125, Italy; f.gobba@unimore.it

**Keywords:** foot pain, physical symptoms, questionnaire, computer

## Abstract

The use of the computers and other technical devices has increased. The aim of our work was to study the possible relation between self-reported foot symptoms and use of computers and cell phones using a questionnaire. The study was carried out as a cross-sectional study by posting a questionnaire to 15,000 working-age Finns. A total of 6121 responded, and 7.1% of respondents reported that they very often experienced pain, numbness, and aches in the feet. They also often experienced other symptoms: 52.3% had symptoms in the neck, 53.5% in had problems in the hip and lower back, and 14.6% often had sleeping disorders/disturbances. Only 11.2% of the respondents thought that their symptoms were connected to the use of desktop computers. We found that persons with symptoms in the feet quite often, or more often, had additional physical and mental symptoms. In future studies, it is important to take into account that the persons with symptoms in the feet may very often have other symptoms, and the use of computers can influence these symptoms.

## 1. Introduction

Foot pain (FP) is a relatively common complaint in the general population: in a large systematic review based on more than 75,000 participants aged 45 years and over, frequent foot pain was reported by 24% of the participants [1]. FP has a significant impact on mobility [2,3] and, more generally speaking, also on quality of life [1,4,5,6].

Menz et al. [7] studied the age and gender differences in disabling foot pain using different definitions of the Manchester Foot Pain and Disability Index (MFPDI), which can be used to determine the prevalence of disabling foot pain. According to Menz et al. [7], exploration of individual MFPDI items indicated that age significantly affected both the pain intensity and functional limitations, with younger people more likely to report their foot pain being worse in the morning and older people more likely to report functional limitations [7].

Working on a computer is considered a typical example of sedentary work (i.e., work that is characterized by long periods of uninterrupted sitting) [8]. This type of work entails various health risks [9,10,11,12]. According Hu et al. [13] a dose-response relationship between health problems and sitting time was as follows: for every 2 h per day increase in sitting time at work, an association of a 7% increase in risk of diabetes and a 5% increase in risk of obesity was observed. At a standard desktop computer workstation, workers are usually working while sitting. Commissaris et al. [9] studied effects of one standing and three dynamic workstations (a treadmill, an elliptical trainer, and a bicycle ergometer) on computer task performance and cognitive function tests. They concluded that (1) performance of most standard office tasks was hardly affected while using a standing or a dynamic workstation; and (2) a computer task that requires fine motor actions of the hands (e.g., mouse pointing and clicking) was affected by the movements at a dynamic or standing workstation. Sitting can also influence symptoms in feet due to pressures of the body and changes in blood circulation, leading to circulatory problems under the waist line.

In recent years, the use of computers and cell phones has been greatly increased both at work and at leisure all around the world. For example, according to the Finnish Statistics Office [14], in 2012, 88% of Finnish households had a computer, 72% owned a laptop, almost all (90%) young- to middle-aged Finnish persons (18–64 years) used the Internet, and 61% of Finnish people aged 66–74 years used the Internet. In the Fourth European Working Condition Survey Report, around 26% of employees worked with a computer either all or almost of the time [15].

Another interesting aspect is that the increase in use of various technical equipment—such as personal computers (PCs), portable computers, notebooks, cell phones, and more recently, also tablets, e-readers, and smartphones—also involves aged persons. As previously mentioned, age is another risk factor for FP [1,7]; therefore, we started a study in 2002 on possible influences of new technical equipment on the health of the working-age population because it was, and still is, important to recognize the possible health effects that can be associated with new technologies. We sent a questionnaire divided into six sections to about 15,000 Finns. The questionnaire included the following sections: Section 1 dealt with background information, such as age, gender, marital status, education, occupation, and home county; Section 2 gauged participants’ familiarity and use of given technical devices at leisure and at work; Section 3 focused on physical loading and ergonomics; Section 4 was concerned with psychological welfare; Section 5 tallied accidents and close-call situations at leisure or at work; and Section 6 was an open-ended question: “Other observations concerning technology and health.” The details of the questionnaire and the results of the ergonomic health aspects and mental symptoms have been reported earlier [16,17,18,19,20,21,22,23,24,25].

### Aim of the Study

The aim of this study was to determine the possible relation between self-reported foot symptoms, including aches, pain, or numbness, and use of computers and cell phones, to analyze how the symptoms are specifically associated with the use of desktop computers, portable computers or mini-computers, and cell phones. As the use of computers and laptops, but also mini-computers and cell phones, frequently enhance sedentary behavior and prolonged sitting time, is it possible to find associations between the use of this technical equipment and users’ foot symptoms? We evaluated the frequency of self-reported symptoms and the frequency of the use of PCs, laptops, minicomputers, and cell phones. The hypothesis is that these new devices may increase the risk of developing foot symptoms related to a variety of conditions, for example, poor posture. We have also considered the relation between self-reported mental symptoms, other physical symptoms, and background information [17,19,20,23].

## 2. Methods

### 2.1. Study Population and Questionnaire

A questionnaire was sent to 15,000 working-age Finns (in October 2002). Here, we only included people aged 18–65. Names and addresses were obtained as a random sample from the Finnish Population Register Centre. The study design was approved by the local Ethical Committee (Pirkanmaa Health District, Finland, decision R02099). The respondents (*n* = 6121) to the questionnaire had to answer items regarding symptoms in different body segments, including the feet, and items concerning the use of computers and portable computers, including laptops, minicomputers, and cell phones. For example, Question Q11 was related to the frequency and type of electronic devices used: “*How often do you use the following equipment or services at work? (a) mobile phone; (b) desktop computer; (c) Internet; (d) electronic commerce; (e) portable computer or mini-computer; (f) teletext; and (g) digital television*” etc.; possible answers were “*cannot say*”, “*not at all*”, “*less than monthly*”, “*monthly*”, “*weekly*”, and “*daily*”. In Question Q13, the subjects had to describe the occurrence of subjective musculoskeletal symptoms in the last 12 months: “*Have you had any ache, pain, or numbness in the following body part during the last twelve months?*” The anatomic localization options of symptoms were “(*a) in wrists and fingers*; *(b) in elbows and forearms*; *(c) in neck*; *(d) in shoulders*; *(e) in the hip and lower back*; *(f) in feet*”, and the frequencies provided were “*cannot say*”, “*not at all*”, “*sometimes*”, “*quite often*”, “*often*”, and “*very often*”. Likewise, in Question Q16 asked participants to describe their mental symptoms during the last 12 months: “*Have you suffered from (a) sleeping disorders/disturbances*; *(b) depression*; *(c) exhaustion at work*; *(d) substance addiction*; *(e) anxiety*; *or (f) fear situations during the last 12 months?*” The frequencies provided were “*cannot say*”, “*not at all*”, “*less than monthly*”, “*monthly*”, “*weekly*”, and “*daily*”.

### 2.2. Statistical Analysis

Subjects were classified into groups according to the frequency of foot symptoms: Group 1, persons who reported aches, pain, or numbness in the feet very often (FSFG1); Group 2, persons who reported the same symptoms often (FSFG2); Group 3, persons who reported symptoms quite often (FSFG3); Group 4, persons experiencing aches, pain, or numbness in the feet sometimes (FSFG4); and Group 5, persons who reported “not at all” for symptoms in the feet (FSFG0).

In the first analysis of the physical symptoms to Question Q13, “(a) in wrists and fingers; (b) in elbows and forearms; (c) in neck; (d) in shoulders; and (e) in hip and lower back” and of the mental symptoms in Question Q16 “(a) sleeping disorders/disturbances; (b) depression; (c) exhaustion at work; (d) substance addiction; (e) anxiety; or (f) fear situations” we used an independent samples Mann-Whitney U-test, because the sample size of groups were limited and the Mann-Whitney U-test is a good method to analyze opinion scales. Mann-Whitney U-test is also a nonparametric test and should be used with data that is not normally distributed.

In the analyses of persons with symptoms in the feet (FSFG 1–4) and without symptoms in the feet not at all (FSFG0), we compared the subgroups: (1) respondents who used a desktop computer daily at work versus nonusers; (2) respondents who used a portable computer or a mini-computer at work daily versus nonusers; (3) female respondents who used a desktop computer at work daily versus nonusers; and (4) male respondents who used a desktop computer at work daily versus nonusers.

In the second analysis, we used persons who experienced symptoms in the feet quite often or more (FSFG 1–3 together). In the analysis, we used an independent samples Mann-Whitney U-test, and we compared the answers to Questions Q13 “(*a) in wrists and fingers*; *(b) in elbows and forearms*; *(c) in neck*; *(d) in shoulders*; and *(e) in hip and lower back*” and Q16 between persons with symptoms in the feet quite often or more (FSFG 1–3 together) and others within subgroups: (1) all respondents in working life; (2) respondents who used a desktop computer daily at work; (3) female respondents who used a desktop computer at work daily; (4) male respondents who used a desktop computer at work daily; and (5) respondents who used a portable computer or a mini-computer at work daily.

In the third analysis, we first classified age groups (under 20, 21–30, 31–40, 41–50, 51–60, and over 60). The statistical analyses consisted of general linear models with the symptoms assigned as target variables. The model factors were as follows: age, gender, daily usage of a desktop computer at work (Q11b), daily usage of a portable computer or mini-computer at work (Q11e), occupation, and two-way interactions of age × gender.

We classified two responses to Question Q11 (“How often do you use the following equipment or services at work?”) “(b) desktop computer” and “(e) portable computer or mini-computer”, so that answers “cannot say”, “not at all”, “less than monthly”, “monthly”, and “weekly” were coded as 0 (no daily use) and the answer “daily” as 1 (daily use). The occupations were classified as follows: “none” was 1, “enterprisers” were 2, “farmers” were 3, “upper-level white-collar workers” were 4, “lower-level white-collar workers” were 5, “blue-collar workers” were 6, “homework, student” was 7, and “other” was 8 in the analyses for Question Q13: “(a) in wrists and fingers; (b) in elbows and forearms; (c) in neck; (d) in shoulders; and (e) in hip and lower back”.

We performed this analysis on (I) persons with symptoms in the feet quite often or more (FSFG 1–3 together); (II) persons with symptoms in the feet very often (FSFG1); (III) persons with symptoms in the feet often (FSFG2); and (IV) persons with symptoms quite often (FSFG3). We have reported earlier on the different analyses of mental symptoms [17]. Therefore, we did not use Question Q16 in the third analysis. In this study, a *p*-value of 0.05 was chosen. All statistical analyses were performed using IBM SPSS Statistics version 23.

## 3. Results

### 3.1. Background Information

In total, 6121 completed questionnaires were collected; thus the response rate was 41%. The mean age ± standard deviation (SD) was 41.3 ± 13.1 years. Table 1 shows the data for mean age ± SD and employment rate for groups FSFG1, FSFG2, FSFG3, FSFG4, and FSFG0.

Table 2 presents background information and the results of all persons with foot symptoms “very often”, women with foot symptoms “very often”, and men with foot symptoms “very often”. FSFG1 included 283 (66.3%) women and 144 (33.7%) men. Table 2 shows also that 33.0% of respondents with symptoms in the feet very often used daily desktop computers at work. Table 2 included only the daily use of different technical devices. It reports the other symptoms of the respondents with symptoms in the feet very often and provides the number of only “very often” answers.

### 3.2. Results of the Mann-Whitney U-Test

Table 3 shows the analyses of physical symptoms in wrists and fingers, elbows and forearms, neck, shoulders, and hip and lower back (Question Q13a–e, respectively). In the analyses of FSFG1 (symptoms experienced very often), there were some significant differences in the comparison between female daily desktop computer users and nonusers in terms of the aches, pain, or numbness in the shoulder symptoms, and between daily laptop users and nonusers in wrist and finger symptoms. In FSFG2 (symptoms experienced often), there was a significant difference in the comparison between male daily portable computer users and nonusers in terms of aches, pain, or numbness in the wrists and fingers. In FSFG3 (symptoms experienced quite often), there were significant differences in the comparison between female daily desktop computer users and nonusers in terms of the aches, pain, or numbness in the neck symptoms. In FSFG4 (symptoms experienced sometimes), there were significant differences in the comparison between male daily desktop computer users and male nonusers in terms of the aches, pain, or numbness in the wrists and fingers. In addition, the comparison of female users (of FSFG4) and female nonusers of desktop computers yielded a significant difference in neck symptoms. In the analysis of FSFG0 (no symptoms), there were significant differences in all other groups except hip and lower back.

Table 4 provides the analyses of questions on mental symptoms: sleeping disorders, depression, exhaustion at work, substance addiction, anxiety, and fear situations (Q16a–f, respectively). In all the data and men’s data (FSFG2), there was a difference in the answers regarding sleep disorders/disturbances, depression, and anxiety between daily desktop computer users and nonusers. In FSFG4, there were significant differences in sleeping disorders/disturbances and exhaustion at work between daily desktop computer users and nonusers.

Table 5 gives the analyses between persons with symptoms in the feet quite often or more and others. In all of the workers’ and daily desktop computer users’ data, there were differences between all in Questions Q13a–e and Q16a–c, e and f. Similarly, there were significant differences between the women’s and men’s data.

### 3.3. General Linear Model (GLM) Results

Table 6 delineates the results of the statistical analyses of answers to Question Q13a–e with the following factors: age, gender, daily usage of a desktop computer at work (Q11b), daily usage of a portable computer or mini-computer at work (Q11e), and occupation. There are no results presented for the analyses of FSFG1 (symptoms very often in feet) because there were no significant differences.

Age had an influence on Question Q13a,b,d in some of the groups. Gender had an influence on Question Q13c,d in some of the groups. Occupation had an influence on Questions Q13e (symptoms in the hip and lower back) in FSFG3 (symptoms in feet quite often). Use of a portable computer had an influence on Questions Q13a (symptoms in wrists and fingers) in some analyses. Some relation can also be seen together with two-way interactions.

## 4. Discussion

### 4.1. Evaluation of Methods

The recruited study population was 15,000 working-age Finns, and we received a large number of responses (6121). Therefore, we could perform analyses on the subgroups of persons with physical symptoms in the feet. In these analyses, 427 responses were used from persons who reported aches, pain, or numbness in the feet very often during the last 12 months. The questionnaire included only three questions regarding symptoms in the feet. Therefore, we were not able to obtain very much information about the respondents’ specific foot symptoms.

The approach applied in this study has some limitations. For example, the questionnaire and questions could have influenced participants, and only those who were active might have sent back the questionnaire. In addition, personal opinions can change quite quickly as technology develops. All participants perhaps did not understand the symptoms in the same manner. We did not ask anything regarding physical load at work. There can also be other factors that influence symptoms at work, for example, sitting or standing positions at workstations.

On the other hand, we are confident that the adopted approach is valid to study a possible relation with the use of computers.

Data were collected in 2002–2003, but there is no reason to suppose that the possible relation between computer use and symptoms has changed. In addition, several workers use similar computers nowadays to what they used to do when we collected the data. Therefore, our data is still valid. Moreover, the use of computers has increased, and many tasks have changed such that workers use computers more. For example, the use of paper has decreased in offices, and workers sit more than in previous years. It is important to evaluate the data and find possible associations.

### 4.2. Evaluation of Results of Persons with Symptoms in Feet “Very Often”

Table 1 shows that in the data of persons with symptoms in the feet (very often), there was a higher prevalence among blue-collar workers, and lower- and upper-level white collar workers, with blue-collar workers being the highest (44.7%). They may have more physical load or standing workstations in their occupations than white collar workers; however, this is speculative, as we did not collect data on physical load or working environments.

Persons with symptoms in the feet (very often) were older and more likely to be retired than other persons. It is possible that their symptoms were the reason why they were not employed any longer. Seventy-four percent of them used a cell phone daily at leisure, and 33% of them used a desktop computer daily at work. Furthermore, only 11.2% of them thought that their symptoms were very often associated with desktop computer use, and 7% of them thought that their symptoms were sometimes associated with desktop computers. It is possible that some of the respondents with symptoms very often in the feet had problems with the ergonomics of the computer; therefore, they had symptoms in the feet. However, it was only a small group of respondents.

The persons with symptoms in the feet very often also had many other physical and mental symptoms (Table 2). For example, 52.3% of them had symptoms in the neck, 53.5% had symptoms in the hip and lower back, and 39.7% had symptoms in the shoulders, which are comparatively higher than in data for all respondents (15.1%, 7.0%, and 8.0%, respectively). Conversely, it is quite easy to understand that persons can have symptoms in different areas of the body. They also reported more sleeping disorders/disturbances, depression, exhaustion at work, anxiety, and fear situations compared with all respondents. For example, 14.6% of them had sleeping disorders/disturbances very often; 11.5% of them had exhaustion at work; and 6.7% had depression very often. All respondents’ values were 3.3%, 2.7%, and 1.7%, respectively. The persons with symptoms in the feet (very often) often also had other physical or mental symptoms.

Women who had symptoms in the feet (symptoms experienced very often) reported more often experiencing other physical symptoms than men (Table 2). Typically, women reported more symptoms than men. For example, Hogg-Johnson et al. [26] have also reported that neck pain was more prevalent among women. Men with symptoms in the feet very often reported fewer additional symptoms. Only in the substance addiction category did men report more symptoms than women.

We found significant differences when we compared persons with symptoms quite often or more often in the feet to the persons who did not report as many symptoms (Table 5). There were many significant differences when we compared persons who used computers daily at work. Computer use can affect persons with symptoms in the feet as well as other physical and mental symptoms. However, there is no difference in substance addiction.

Table 6 reveals that we found significant differences, for example, in age, gender, and occupation. In FSFG1–3 together (symptoms in the feet very often, often, and quite often), respondents with symptoms were older than others. In FSFG3 (foot symptoms quite often), occupation had a significant impact on the symptoms in the hip and lower back. It is possible that persons with symptoms in the feet (FSFG3) also have other causes for their symptoms. There are also differences when comparing the use of portable computers. However, only 17 respondents with foot symptoms very often used portable computers daily at work. Therefore, it is not possible to do draw strong conclusions regarding laptop use.

Based on our results, it is possible that the use of computers influences persons’ symptoms in the feet, but there are also other possible explanations, such as age and mental symptoms. It is also possible that sitting workstations can influence foot symptoms because workers typically sit and use desktop computers. We found some differences with computer users and nonusers. However, only 11.2% of the respondents (with foot symptoms very often) thought that their symptoms were connected to the use of desktop computers.

We did not inquire about physical load at work, which could also be one explanation. Eklöf et al. [27] and Pransky et al. [28] have found that stress is also a common problem in working life that is related to psychosocial factors, and that it may be associated with musculoskeletal complaints. Their findings support our results. It is also an important finding that persons with symptoms very often in the feet had many other physical and mental symptoms very often; for example, 14.6% of them had sleeping disorders/disturbances. An aspect that cannot be ignored is that the use of new technologies, such as portable computers and cell phones, may interfere with psychosocial factors, (e.g., increasing workload).

Our hypothesis was that these new devices may increase the risk of developing foot pain related to poor postures (for example) or to other factors, and we analyzed the relation among self-reported mental symptoms, other physical symptoms, and background information. We found some significant differences in groups of persons with self-reported aches, pain, or numbness in the feet very often, often, quite often, and sometimes (e.g., symptoms in the wrists and fingers, in the neck, and in the shoulders). However, we did not uncover significant differences in all groups. We also found significant differences in other symptoms and background information between persons with symptoms in the feet quite often or more and others using different subgroups. In general, we can accept our hypothesis.

## 5. Conclusions

The results of this questionnaire study show that 7.1% of Finnish workers experience pain, numbness, and aches in the feet very often. Thirty-three percent of them used a desktop computer at work daily. When comparing persons with symptoms in the feet quite often or more often compared to others, we found that they had increased additional physical and mental symptoms. There were also significant differences in use of different computers at work. In the future, it is essential to take into account that the persons with symptoms in the feet can very often have other symptoms (e.g., sleeping disorders/disturbances). Additionally, the usage of different computers can influence their symptoms.

## Figures and Tables

**Table 1:** Data for mean age ± SD and employment rate of all and groups FSFG1, FSFG2, FSFG3, FSFG4, and FSFG0.

Groups with or without Symptoms in the Feet	Number (%)	Mean Age ± SD	Employment Rate % *
All respondents of 15,000 recruited	6121 (41% of all)	41.3 ± 13.1	71.4%
Symptoms very often in the feet, FSFG1	427 (7.1%)	47.4 ± 12.3	61.9%
Symptoms often in the feet, FSFG2	469 (7.8%)	44.9 ± 13.2	67.9%
Symptoms quite often in feet, FSFG3	616 (10.2%)	42.6 ± 13.8	71.4%
Symptoms sometimes in feet, FSFG4	2136 (35.3%)	40.7 ± 13.0	74.5%
Symptoms not at all, FSFG0	2362 (39.1%)	39.4 ± 12.5	76.8%
Missing	38 (0.6%)	-	-

* Valid percent.

**Table 2:** A summary of background information and the results of persons (women and men) with foot symptoms “very often”, the daily usage of different devices/computers at work and at leisure, mental symptoms, and experienced pain, numbness, or aches (number of positive answers of “very often”).

Topics of Questions and Choices	Women %	Men %	Total %
	(*n* = 283)	(*n* = 144)	(*n* = 427)
**Marital status**			
single (*n* = 60)	12.0	18.1	14.1
married or live-in (*n* = 303)	69.3	74.3	71.0
divorced (*n* = 50)	13.8	7.6	11.7
widow or widower (*n* = 14)	4.9	0.0	3.3
**Education**			
comprehensive school (*n* = 134)	29.8	34.7	31.5
matriculation (*n* = 25)	6.7	4.2	5.9
vocational school (*n* = 143)	31.6	37.5	33.6
vocational high school (*n* = 96)	25.9	16.0	22.5
university (*n* = 28)	6.0	7.6	6.6
**Occupation**			
none (*n* = 4)	0.4	2.1	0.9
enterpriser (*n* = 34)	6.4	11.3	8.0
farmer (*n* = 25)	6.8	4.2	5.9
upper-level white-collar workers * (*n* = 40)	8.5	11.3	9.5
lower-level white-collar workers ** (*n* = 85)	23.8	12.7	20.1
blue-collar workers *** (*n* = 189)	41.3	51.4	44.7
homework, student (*n* = 18)	6.0	0.7	4.3
other (*n* = 28)	6.8	6.3	6.6
**Use at leisure (daily)**			
mobile phone (*n* = 314)	72.4	75.7	73.5
desktop computer (*n* = 90)	20.8	21.5	21.1
internet (*n* = 64)	13.8	17.4	15.0
portable computer or mini-computer (*n* = 15)	2.5	5.6	3.5
**Use at work (daily)**			
mobile phone (*n* = 132)	28.6	35.4	30.9
desktop computer (*n* = 141)	38.2	22.9	33.0
internet (*n* = 72)	18.4	13.9	16.9
portable computer or mini-computer (*n* = 17)	3.9	4.2	4.0
**Experienced pain, numbness or aches**			
in wrists or fingers (*n* = 138)	35.7	28.3	33.3
in elbows or forearms (*n* = 91)	23.9	18.9	22.3
in neck (*n* = 220)	59.2	38.1	52.3
in shoulders (*n* = 165)	45.2	28.5	39.7
in hip and lower back (*n* = 224)	57.3	45.7	53.5
**Mental symptoms**			
sleeping disorders/disturbances (*n* = 61)	15.9	12.0	14.6
depression (*n* = 28)	6.9	6.4	6.7
exhaustion at work (*n* = 47)	12.5	9.4	11.5
substance addiction (*n* = 6)	0.7	2.9	1.5
anxiety (*n* = 23)	5.8	5.0	5.6
fear situations (*n* = 14)	3.7	2.9	3.4

* Administrative or managerial duties, designing, research, teaching; ** clerical duties and supervision; *** industrial workers, distributive and service trade.

**Table 3:** Comparison between computer users and nonusers with an independent samples Mann-Whitney U-test analysis for Question Q13a–e, using data of the persons with symptoms in feet very often, often, quite often, sometimes, and not at all.

Q13: Have You Had an Ache, Pain or Numbness in the Following Body Part during the Past 12 Months?					
Participants	Wrists and Fingers	Elbows and Forearms	Neck	Shoulders	Hip and Lower Back
**Persons with symptoms in feet very often (FSFG1, *n* = 427)**					
User vs. nousers	0.871	0.474	0.947	0.185	0.745
Laptop users vs. nousers	0.046 **	0.069	0.829	0.333	0.698
Female users vs. nousers	0.815	0.239	0.357	0.013 **	0.901
Male users vs. nousers	0.870	0.809	0.578	0.282	0.625
**Persons with symptoms in feet often (FSFG2, *n* = 469)**					
Users—nousers	0.513	0.596	0.806	0.629	0.252
Laptop users vs. nousers	0.214	0.478	0.350	0.565	0.852
Female users vs. nousers	0.376	0.175	0.725	0.858	0.478
Male users vs. nousers	0.019 **	0.324	0.463	0.219	0.335
**Persons with symptoms in feet quite often (FSFG3, *n* = 616)**					
Users vs. nousers	0.860	0.122	0.324	0.925	0.817
Laptop users vs. nousers	0.861	0.862	0.311	0.852	0.314
Female users vs. nousers	0.652	0.469	0.043 **	0.204	0.644
Male users vs. nousers	0.387	0.122	0.239	0.054	0.328
**Persons with symptoms in feet sometimes (FSFG4, *n* = 2136)**					
Users vs. nousers	0.032 **	0.324	<0.001 **	0.038 **	0.169
Laptop users vs. nousers	0.171	0.956	0.150	0.745	0.613
Female users vs. nousers	0.313	0.761	0.002 **	0.082	0.291
Male users vs. nousers	0.046 **	0.077	0.033 **	0.423	0.379
**Persons without symptoms in feet (FSFG0, *n* = 2362)**					
Users vs. nousers	0.010 **	0.068	<0.001 **	0.010 **	0.288
Laptop users vs. nousers	0.879	0.454	0.618	0.041 **	0.811
Female users vs. nousers	0.012 **	0.003 **	0.001 **	0.016 **	0.059
Male users vs. nousers	0.286	0.437	0.004 **	0.320	0.642

** Significant at *p* < 0.05; users = workers who use a desktop computer daily at work; laptop users = workers who use daily a portable computer or a mini-computer at work.

**Table 4:** Comparison between computer users and nonusers with an independent samples Mann-Whitney U-test analysis for Question Q16 using data of the persons with symptoms in the feet very often, often, quite often, and sometimes.

Q16: Have You Suffered from One of the Following Symptoms in the Past 12 Months?						
Participants	Sleeping Disorders/Disturbances	Depression	Exhaustion at Work	Substance Addiction	Anxiety	Fear Situations
**Persons with symptoms in feet very often (FSFG1)**						
Users vs. onusers	0.134	0.909	0.196	0.439	0.110	0.579
Laptop users vs. nonusers	0.160	0.297	0.413	0.542	0.564	0.116
Female users vs. nonusers	0.243	0.891	0.064	0.750	0.522	0.848
Male users vs. nonusers	0.420	0.717	0.501	0.620	0.052	0.185
**Persons with symptoms in feet often (FSFG2)**						
Users vs. nonusers	0.001 **	0.372	0.303	0.949	0.756	0.644
Laptop users vs. nonusers	0.068	0.492	0.571	0.430	0.248	0.680
Female users vs. nonusers	0.065	0.673	0.921	0.368	0.159	0.489
Male users vs. nonusers	0.003 **	0.049 **	0.164	0.351	0.022 **	0.052
**Persons with symptoms in feet quite often (FSFG3)**						
Users vs. nonusers	0.685	0.738	0.762	0.919	0.336	0.903
Laptop users vs. nonusers	0.523	0.598	0.693	0.518	0.615	0.145
Female users vs. nonusers	0.791	0.198	0.630	0.764	0.578	0.806
Male users vs. nonusers	0.317	0.285	0.957	0.608	0.459	0.604
**Persons with symptoms in feet sometimes (FSFG4)**						
Users vs. nonusers	0.043 **	0.200	0.008 **	0.481	0.501	0.374
Laptop users vs. nonusers	0.986	0.983	0.229	0.460	0.658	0.840
Female users vs. nonusers	0.273	0.792	<0.001 **	0.815	0.936	0.575
Male users vs. nonusers	0.106	0.114	0.903	0.453	0.418	0.460

** *p* < 0.05, asymptotic significance; users = workers who use a desktop computer daily at work; laptop users = workers who use daily a portable computer or a mini-computer at work.

**Table 5:** Comparison between persons with symptoms in the feet quite often or more and others with an independent samples Mann-Whitney U-test analysis for Questions Q13a–e and Q16, using data of the persons with symptoms in feet quite often or more often and without.

**Q13: Have You Had an Ache, Pain or Numbness in the Following Body Part during the Past 12 Months?**						
**Participants**	**Wrists and Fingers**	**Elbows and Forearms**	**Neck**		**Shoulders**	**Hip and Lower Back**
All workers	<0.001 **	<0.001 **	<0.001 **		<0.001 **	<0.001 **
Daily desktop computer users	<0.001 **	<0.001 **	<0.001 **		<0.001 **	0.001 **
Female desktop computer users	<0.001 **	<0.001 **	<0.001 **		<0.001 **	<0.001 **
Male desktop computer users	<0.001 **	<0.001 **	<0.001 **		<0.001 **	<0.001 **
Laptop users	<0.001 **	<0.001 **	<0.001 **		<0.001 **	<0.001 **
**Q16: Have You Suffered from One of the Following Symptoms in the Past 12 Months?**						
**Participants**	**Sleeping Disorders/Disturbances**	**Depression**	**Exhaustion at Work**	**Substance Addiction**	**Anxiety**	**Fear Situations**
All workers	<0.001 **	<0.001 **	<0.001 **	0.291	<0.001 **	<0.001 **
Daily desktop computer users	<0.001 **	<0.001 **	<0.001 **	0.302	<0.001 **	<0.001 **
Female desktop computer users	<0.001 **	<0.001 **	<0.001 **	0.506	<0.001 **	<0.001 **
Male desktop computer users	<0.001 **	<0.001 **	<0.001 **	0.255	<0.001 **	0.001 **
Daily laptop users	<0.001 **	<0.001 **	<0.001 **	0.254	0.003 **	<0.001 **

** *p* < 0.05, asymptotic significance; users = workers who use a desktop computer daily at work; laptop users = workers who use daily a portable computer or a mini-computer at work.

**Table 6:** The results (type III sum of squares and significance (Sig.) of statistical analyses for Question Q13a–e, using data of the persons with symptoms in the feet very often, often, and quite often.

Q13: Have You Had an Ache, Pain or Numbness in the Following Body Part during the Past 12 Months?					
Source of Variation	Wrists and Fingers	Elbows and Forearms	Neck	Shoulders	Hip and Lower Back
	Sig.	Sig.	Sig.	Sig.	Sig.
Persons with symptoms feet quite often or more (FSFG 1–3)					
Age	0.018 **	0.001 **	n.s.	0.017 **	n.s.
Gender	n.s.	n.s.	<0.001 **	0.014 **	n.s.
Use of a desktop computer ^1^	n.s.	n.s.	n.s.	n.s.	n.s.
Use of a portable computer ^2^	0.008 **	n.s.	n.s.	n.s.	n.s.
Occupation	n.s.	n.s.	n.s.	n.s.	n.s.
*Two-way interaction*					
Age × gender	0.021**	n.s.	n.s.	n.s.	n.s.
Persons with symptoms in feet often (FSFG2)					
Age	0.024 **	n.s.	n.s.	n.s.	n.s.
Gender	n.s.	n.s.	<0.001 **	n.s.	n.s.
Use of a desktop computer ^1^	n.s.	n.s.	n.s.	n.s.	n.s.
Use of a portable computer ^2^	0.043 **	n.s.	n.s.	n.s.	n.s.
Occupation	n.s.	n.s.	n.s.	n.s.	n.s.
*Two-way interaction*					
Age × gender	0.016 **	n.s.	n.s.	n.s.	n.s.
Persons with symptoms in feet quite often (FSFG3)					
Age	n.s.	n.s.	n.s.	n.s.	n.s.
Gender	n.s.	n.s.	0.001 **	n.s.	n.s.
Use of a desktop computer ^1^	n.s.	n.s.	n.s.	n.s.	n.s.
Use of a portable computer ^2^	n.s.	n.s.	n.s.	n.s.	n.s.
Occupation	n.s.	n.s.	n.s.	n.s.	0.007 **
*Two-way interaction*					
Age × gender	n.s	n.s.	n.s.	n.s.	n.s.

** Significant at *p* < 0.05; ^1^ daily use of a desktop computer at work; ^2^ daily use of a portable computer or mini-computer at work; n.s.: not significant.
